# A Convergent Functional Genomics Analysis to Identify Biological Regulators Mediating Effects of Creatine Supplementation

**DOI:** 10.3390/nu13082521

**Published:** 2021-07-23

**Authors:** Diego A. Bonilla, Yurany Moreno, Eric S. Rawson, Diego A. Forero, Jeffrey R. Stout, Chad M. Kerksick, Michael D. Roberts, Richard B. Kreider

**Affiliations:** 1 Research Division, Dynamical Business & Science Society—DBSS International SAS, Bogotá 110861, Colombia; luzyuranymoreno@gmail.com; 2 Research Group in Biochemistry and Molecular Biology, Universidad Distrital Francisco José de Caldas, Bogotá 110311, Colombia; 3 Research Group in Physical Activity, Sports and Health Sciences (GICAFS), Universidad de Córdoba, Montería 230002, Colombia; 4 kDNA Genomics^®^, Joxe Mari Korta Research Center, University of the Basque Country UPV/EHU, 20018 Donostia-San Sebastián, Spain; 5 Department of Health, Nutrition and Exercise Science, Messiah University, Mechanicsburg, PA 17055, USA; erawson@messiah.edu; 6 Professional Program in Sport Training, School of Health and Sport Sciences, Fundación Universitaria del Área Andina, Bogotá 111221, Colombia; dforero41@areandina.edu.co; 7 Physiology of Work and Exercise Response (POWER) Laboratory, Institute of Exercise Physiology and Rehabilitation Science, University of Central Florida, Orlando, FL 32816, USA; jeffrey.stout@ucf.edu; 8 Exercise and Performance Nutrition Laboratory, School of Health Sciences, Lindenwood University, Saint Charles, MO 63301, USA; ckerksick@lindenwood.edu; 9 School of Kinesiology, Auburn University, Auburn, AL 36849, USA; mdr0024@auburn.edu; 10 Edward via College of Osteopathic Medicine, Auburn, AL 36849, USA; 11 Exercise & Sport Nutrition Laboratory, Human Clinical Research Facility, Texas A&M University, College Station, TX 77843, USA; rbkreider@tamu.edu

**Keywords:** creatine kinase, systems biology, bioinformatics, MAP kinase signaling system, sodium-chloride-dependent neurotransmitter symporters, signal transduction

## Abstract

Creatine (Cr) and phosphocreatine (PCr) are physiologically essential molecules for life, given they serve as rapid and localized support of energy- and mechanical-dependent processes. This evolutionary advantage is based on the action of creatine kinase (CK) isozymes that connect places of ATP synthesis with sites of ATP consumption (the CK/PCr system). Supplementation with creatine monohydrate (CrM) can enhance this system, resulting in well-known ergogenic effects and potential health or therapeutic benefits. In spite of our vast knowledge about these molecules, no integrative analysis of molecular mechanisms under a systems biology approach has been performed to date; thus, we aimed to perform for the first time a convergent functional genomics analysis to identify biological regulators mediating the effects of Cr supplementation in health and disease. A total of 35 differentially expressed genes were analyzed. We identified top-ranked pathways and biological processes mediating the effects of Cr supplementation. The impact of CrM on miRNAs merits more research. We also cautiously suggest two dose–response functional pathways (kinase- and ubiquitin-driven) for the regulation of the Cr uptake. Our functional enrichment analysis, the knowledge-based pathway reconstruction, and the identification of hub nodes provide meaningful information for future studies. This work contributes to a better understanding of the well-reported benefits of Cr in sports and its potential in health and disease conditions, although further clinical research is needed to validate the proposed mechanisms.

## 1. Introduction

Creatine (Cr), or alpha-methylguanidinoacetic acid (PubChem CID: 586), and its phosphorylated form, phosphocreatine (PCr), are essential molecules for the optimal functioning of tissues with high and fluctuating energy demands [1]. They provide an evolutionary advantage via several creatine kinase (CK) isozymes that functionally connect places of adenosine triphosphate (ATP) synthesis with sites of ATP consumption (the CK/PCr system) [2]; therefore, Cr and PCr are physiologically essential for life through a rapid and localized support of energy- and mechanical-dependent processes (i.e., cell survival, growth, proliferation, differentiation, and migration or motility) [3,4]. For a recent and comprehensive review of the Cr metabolism, please refer to [3].

The CK/PCr system can be enhanced through supplementation with creatine monohydrate (CrM), which is the most studied, safe, and effective nutritional supplement to optimize physical performance [5,6,7,8], with potential benefits in health and disease [9,10,11,12,13,14,15,16,17,18,19]. It seems that the elevation of intracellular PCr concentration causes a greater capacity for phosphagens to contribute to energy metabolism, while working to reduce the accumulation of Pi and H^+^ and improving Ca^2+^ handling as important mediators of fatigability in young and older adults [20,21,22,23]. This has previously been reported in vivo and in vitro after CrM supplementation [24,25,26,27,28,29]. Notwithstanding, a cellular environment rich in high-energy phosphates might also trigger downstream signaling pathways that are sensitive to energy changes by activating secondary messengers and protein kinases [30,31]. In this sense, several clinical trials [32,33,34,35] and research in animal models [36,37,38] have revealed that CrM administration regulates the expression of particular genes or proteins using low-throughput screening. In accordance with Kontou et al. [39], individual experiments can only identify targeted regulators (based on prior knowledge), with limited comprehension of how the biological system works. As a result, data integration from multiple experimental studies and public repositories is necessary to understand the function of biological entities (e.g., genes, proteins) and their expression patterns under certain conditions. Additionally, ‘omics’ technologies in conjunction with the advance of bioinformatics tools allow for data integration and the extraction of biologically relevant information, such as identifying biomarkers and regulatory components within a network [40]. Consequently, the use of a systems biology approach guarantees identification, at a systems scale, of the molecular signatures of cellular processes, molecular interactions, and relevant metabolic pathways present in the complex physiological responses or diseases with multi-factorial underpinnings [41,42]. Among the different approaches, convergent functional genomics (CFG) is an interesting methodological approach for the integrative analysis of molecular mechanisms by combining multiple lines of genomic evidence from different species [43]. CFG takes advantage of the conserved nature of metabolic circuits between several species (e.g., rodents and humans) [44] to provide relevant information about the structural and functional changes within the cell when there is not enough available data or the experiments are difficult, if not impossible, to conduct in humans (e.g., those in brain tissue) [45]. While human data increase the clinical relevance (specificity), animal model data increase the ability to identify the signal (sensitivity). Combined together, we enhance our ability to distinguish signals from noise, even with limited cohorts and datasets [46]; therefore, CFG is useful for identifying novel candidate genes and pathways for specific phenotypes [47,48,49,50] and compound-mediated gene regulation [51,52]. It is necessary to point out the complementary features of low- and high-throughput analysis, given that subsequent validation of the identified metabolic hubs requires the high sensitivity, lower noise, and reproducibility of low-throughput techniques (e.g., post-transcriptional regulation assessment, targeted-molecules expression, protein–protein interactions) [53].

In the context of novel models of physiological regulation, the concept of allostasis was developed. This highlights the importance of the anticipation of needs (such as the timely provision of energy and adequate environmental conditions) for the functional and structural stability of cells through adaptive changes [54]. Although efforts have been made to integrate the different points of metabolic regulation to explain the positive effects of CrM supplementation on physical performance [55,56,57] and health or therapeutic benefits [58,59,60], no systems biology analysis has been performed to date. Readers are encouraged to refer to the comprehensive reviews in the Special Issue on “Creatine Supplementation for Health and Clinical Diseases” to learn more about the effects of CrM supplementation [9]. The use of a systems biology approach might contribute to better comprehension of the molecular, cellular, tissue, and systemic effects of CrM and its applications to health and disease; thus, the aim of this study was to perform for the first time a CFG analysis to identify relevant pathways and biological processes mediating the effects of Cr supplementation in health and disease. This secondary analysis of the available data on differentially expressed genes after CrM administration in humans and mice will provide meaningful information for future studies.

## 2. Methods

### 2.1. Functional Genomic Analysis

#### 2.1.1. Search and Sources of Evidence

In order to collect the gene expression data, two public repositories (NCBI Gene Expression Omnibus (GEO: http://www.ncbi.nlm.nih.gov/geo (accessed on 14 January 2021)) and the ArrayExpress Archive (https://www.ebi.ac.uk/arrayexpress/) (accessed on 14 January 2021) were searched following the PRISMA statement guidelines [61] and international recommendations [62]. The following Boolean algorithm was used in the GEO repository: *(“creatine”[MeSH Terms] OR creatine monohydrate[All Fields]) AND supplementation[All Fields]*, while the free term “creatine” was used in the ArrayExpress Archive. Repositories were accessed on 14 January 2021, although an updated search was conducted prior to manuscript submission. We also contacted corresponding authors (e-mail communication) to obtain missing raw data when no record was found in the repositories.

#### 2.1.2. Eligibility Criteria

The inclusion of gene expression data for the CFG analysis was restricted to the following: (1) original experimental studies that screened for genes differing between Cr and controls; (2) raw data deposited in the NCBI GEO or the ArrayExpress Archive; (3) expression data obtained from any tissue or cell; (4) human or mouse experimental models. The search excluded data obtained from the combination of Cr with other compounds. Information was extracted from each identified record and reported in a table, including the organism, reference, GEO accession number, sample type, numbers of cases and controls, and platform.

#### 2.1.3. Analysis of Differentially Expressed Genes and Convergence

The GEO2R web application [63] was used to identify differentially expressed (DE) genes in the datasets for human and mouse models (freely available at http://www.ncbi.nlm.nih.gov/geo/geo2r/, accessed on 18 January 2021). For the analysis with the GEO2R tool, samples from Cr treatment were taken as the experimental group, while untreated samples were taken as the control group. GEO2R provides the following summary statistics as generated by ‘limma’, which performs the top table computation to extract a table of the top-ranked genes, including the adjusted *p*-values and raw *p*-values; moderated t-statistics, B-statistics, or log-odds that the gene is differentially expressed; the log2-fold changes between pairs of experimental conditions; and moderated F-statistics (which combines the t-statistics for all the pair-wise comparisons into an overall test of significance for that gene) [64].

A CFG approach was used to identify a list of candidate genes with multiple lines of evidence from humans and mouse models. After the GEO2R analysis, the gene names were extracted from the differentially expressed genes based on the adjusted *p*-values using the Benjamini–Hochberg method. An online tool developed by Bioinformatics and Evolutionary Genomics at VIB/UGent (Gent, Belgium) (http://bioinformatics.psb.ugent.be/webtools/Venn) was used to identify convergence in the list of genes from these datasets (accessed on 29 January 2021).

#### 2.1.4. Functional Enrichment Analysis

Gene Ontology (GO) enrichment analysis of the list of genes from the convergent analysis was performed with the gene set enrichment analysis tool for mammalian gene sets from Enrichr, which is a comprehensive resource for curated gene sets that was published in 2013 [65] (http://amp.pharm.mssm.edu/Enrichr, accessed on 12 May 2021). An exploratory analysis with the DAVID tool (https://david.ncifcrf.gov/, accessed on 12 May 2021) showed similar results to top terms sorted by p-value ranking using the Enrichr platform, an outcome that was somewhat expected given the use a scoring method similar to both the hypergeometric test and Fisher’s exact test; however, the advantages of Enrichr over other tools are its comprehensiveness, ease of use, interactive visualization of the results, and the calculation of a combined score, which has been demonstrated to recover more of the ‘correct’ terms compared with the other methods (e.g., the over-representation analysis, the Jaccard distance, or the number of overlapping genes) [66]. Enrichr also retrieves the computational predictions of interactions between the list of genes from the convergent analysis and small non-coding microRNAs (known as miRNAs) using the miRTarBase library [67], which contains experimentally validated miRNA–mRNA interactions. We reported the categories ranked as statistically significant based on adjusted *p*-values using an inherent z-score permutation background correction on Fisher’s exact test. The bioinformatics tools were accessed from 20 February to 12 May 2021.

#### 2.1.5. Upstream Regulatory Pathway Analysis

We utilized the differentially expressed genes from the CFG analysis to infer the upstream regulatory networks using the computational pipeline of the eXpression2Kinases (X2K) Web (freely available at https://amp.pharm.mssm.edu/X2K/, accessed on 12 May 2021) [68]. X2K Web is an enhanced algorithm that performs an enrichment analysis to prioritize transcription factors that most likely regulate the observed changes in mRNA expression (ChEA and PWM), which was previously validated by Chen et al. [42]. It then utilizes known protein–protein interactions (PPIs) to connect the identified transcription factors to form a subnetwork. Finally, kinase enrichment analysis (KEA) is performed to prioritize protein kinases known to phosphorylate substrates within the subnetwork of transcription factors and the intermediate proteins that connect them. Top kinases and regulatory proteins (ranked by hypergeometric *p*-values, which indicate unusual differential expression in the database) were contrasted to individual low-throughput experimental reports available in the literature.

## 3. Findings

### 3.1. Selection of Gene Expression Datasets

**Figure 1:**
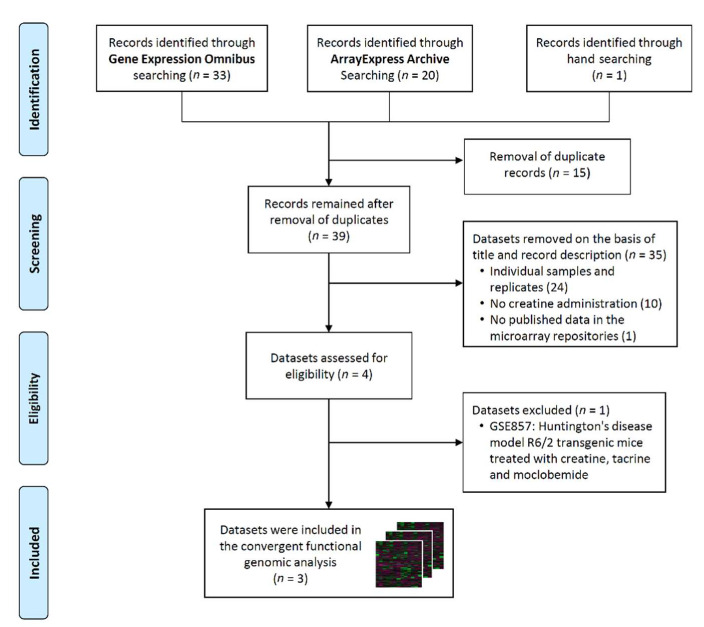
PRISMA flow diagram.

After retrieving the records using the Boolean algorithm, we obtained 33 datasets from the GEO database, 20 from the ArrayExpress Archive, and one from the hand searching process in Google Scholar. The expression data for one study that assessed changes in muscle transcriptome after Cr supplementation during knee immobilization in healthy young men [69] was not published within a public repository (unsuccessful e-mail communication). After the assessment for inclusion criteria, only four records were suitable for eligibility, although one study was excluded from the analysis because a treatment combination with Cr, tacrine, and moclobemide was carried out. The overall procedure for data extraction is shown in the PRISMA flow chart (Figure 1).

We downloaded the gene expression datasets GSE7877 [70] (human), GSE5140 [71] and GSE42356 [72] (mouse) from the GEO. Table 1 shows the characteristics of the studies and datasets that were included in our CFG analysis.

### 3.2. Analysis of Differentially Expressed Genes

After the analysis with the GEO2R tool, the differentially expressed genes were selected using the adjusted *p* < 0.05 as the cut-off. Subsequently, these lists of genes were filtered for duplicates and empty gene names to retrieve 112 genes from GSE5140 (Table S1) and 11 genes from GSE42356 (Table S2). The available expression profile analyzed by Safdar et al. [70] with 34 genes from GSE7877 was used for subsequent analysis (Table S3).

### 3.3. Analysis of Convergence between Datasets

**Figure 2:**
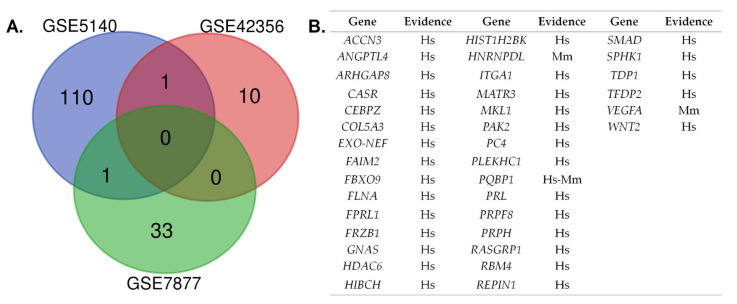
Convergent analysis of the differentially expressed genes obtained from the three datasets: (**A**) Venn diagram comparing differentially expressed genes identified in the included datasets (adjusted *p* < 0.05); (**B**) list of genes derived from the convergent functional genomics analysis in human and mouse models. Hs: *Homo sapiens*; Mm: *Mus musculus*.

The results of the convergent analysis are shown in Figure 2. The human and mice datasets converged in polyglutamine-binding protein 1 (*PQBP1*). This gene encodes a scaffold protein involved in the regulation of transcription, alternative mRNA splicing (via spliceosome), innate immunity, and neuron projection development (UniProtKB-O60828). The list of genes used for the subsequent enrichment analysis encompassed two genes from the converged results and 33 differentially expressed genes from the GSE7877 dataset (*Homo sapiens*).

### 3.4. Functional Enrichment Analysis

Functional annotation revealed that the 35 selected genes are involved in the regulation of apoptosis, proliferation–differentiation, and replication–transcription processes. Table 2 shows the functional annotation GO terms (biological process, molecular function, and cellular component) and the prediction analysis of interactions between the input list and miRNAs.

### 3.5. Upstream Regulatory Pathway Analysis

**Figure 3:**
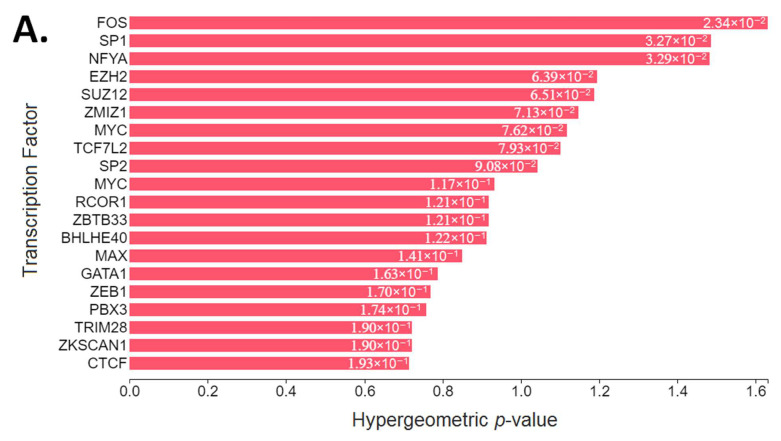

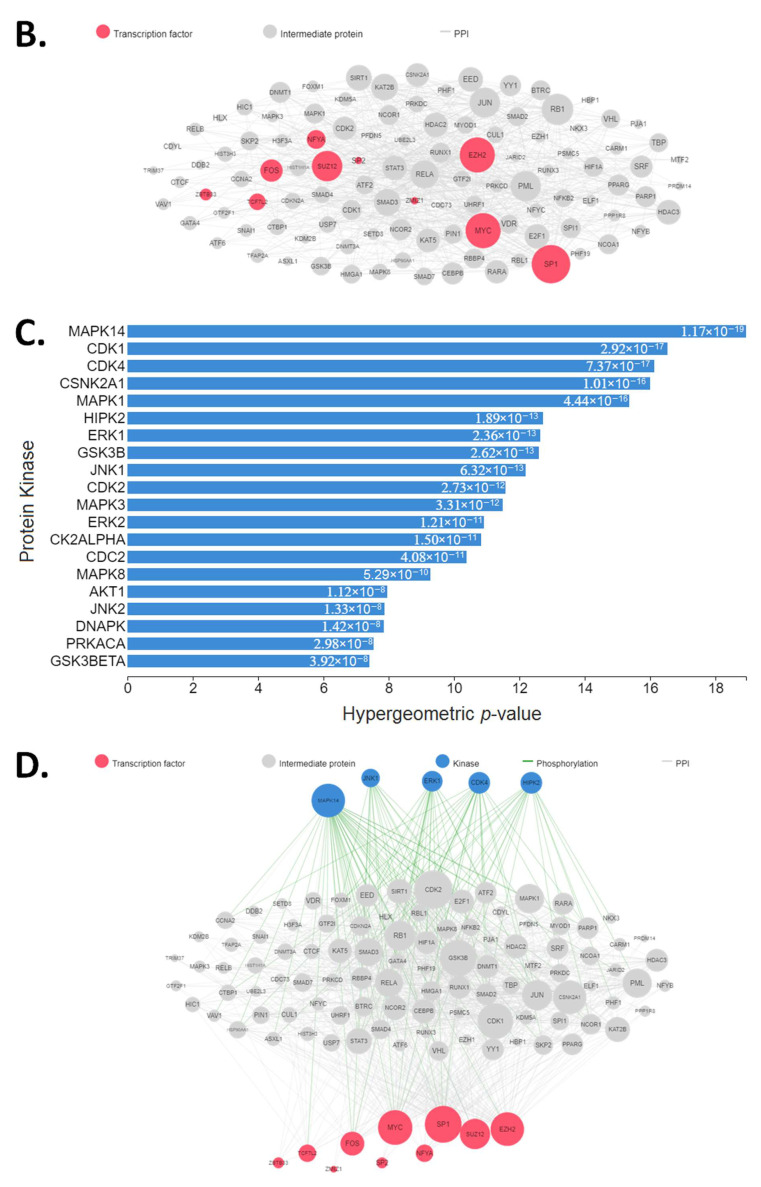
Upstream regulatory pathway analysis of the 35 selected differentially expressed genes: (**A**). transcription factor enrichment analysis; (**B**) protein–protein interaction expansion; (**C**) kinase enrichment analysis; (**D**) eXpression2Kinases network. Figures were obtained from the X2K Web (https://amp.pharm.mssm.edu/X2K/, accessed on 12 May 2021) after running the upstream pathway analysis of the selected genes as the input gene list.

The upstream pathway analysis resulted in the proto-oncogene c-Fos (FOS), specificity protein 1 (SP1), nuclear transcription factor Y subunit alpha (NFYA), the enhancer of zeste homolog 2 (EZH2) and suppressor of zeste 12 (SUZ12) complex, zinc finger MIZ-type containing 1 (ZMIZ1), Myc proto-oncogene protein (MYC), protein max (MAX, also known as Myc-associated factor X), and erythroid transcription factor (GATA1) as the top transcription factors that most likely regulate the observed changes in gene expression after Cr administration. Our kinase enrichment analysis showed that mitogen-activated protein kinase 14 (MAPK14, also known as p38), cyclin-dependent kinases (CDKs), casein kinase II subunit alpha (CSNK2A1, also known as CK2A1), extracellular signal-regulated kinases (ERKs), protein kinase B (Akt/PKB), and c-Jun N-terminal kinases (JNKs) are the top signaling pathways that activate kinases known to phosphorylate substrates within our subnetwork of transcription factors and intermediate proteins (Figure 3).

## 4. Discussion

From a systems biology perspective, the analysis of interactions and characteristics of the system’s components (e.g., molecular system bioenergetics, genome-wide gene-expression profiling, and pathway identification) facilitates the deciphering of the action mechanisms of various system-level properties and biological functions [73]. The CFG approach has been described as a Bayesian way of cross-validating biological findings while reducing uncertainty. In addition, the bioinformatics enrichment of groups of converged genes leads to insights into pathways and mechanisms that may be involved in different phenotypes [74]. This methodology has been successfully utilized, even with limited size cohorts and datasets, in the study of gene associations with chronic fatigue syndrome [50]; biomarker identification for suicidality [48], attention-deficit–hyperactivity disorder [49], stress-related psychiatric disorders [75], and mood disorders [52]; and the discovery of novel candidate genes and signaling pathways for epileptogenesis [47] and retinol or retinoic acid exposure [51]. In order to understand the underlying biological processes and mechanisms mediating the effects of Cr administration, we performed a CFG analysis on differentially expressed genes in humans and mice for the first time. Under the allostasis paradigm (that is, adaptation to changes through mechanisms that alter the set point of metabolic or physiological variables) [76], the CK/PCr system should be seen as an essential mechanism for life (cell survival, growth, proliferation, differentiation, and migration or motility). We previously suggested that the CK/PCr system works as a dynamic biosensor of chemomechanical energy transduction [3] with ‘concurrent reactions with exchange motifs’ [77] that might account for the wide range of diseases after alterations in intracellular Cr concentrations. In agreement with the established regulation of cellular allostasis through a complex balance of subcellular energy production and cellular mechanics [78], our CFG and enrichment analysis showed several biological regulators related to energy metabolism (extra- and intramitochondrial pathways) and cytoskeletal machinery (motor and cytolinker proteins). This makes the CK/PCr system a fractal model that can be used to exemplify the cytoskeleton-mediated, energy-driven, mechanoadaptative processes of the cells. This is the first time an integrative approach has been implemented to elucidate how the enhanced Cr metabolism (via CrM administration) is directly involved in the cellular adaptations through a complex balance of subcellular energy production and cellular mechanics.

### 4.1. Biological Pathways Mediating Effects

The functional annotation of the 35 selected differentially expressed genes after CrM administration showed that these are involved in anti-apoptotic processes, cell differentiation, and positive regulation of protein phosphorylation cascades. It must be noted that these genes perform functions throughout the cell (nuclear origin of replication recognition complex (nucleus), U5 snRNP (transcription complex), and endosome lumen (vesicles)) through either receptor activation and secondary messengers (G-protein-coupled receptor binding, Rac GTPase binding, and polyubiquitin modification-dependent protein binding) or direct regulation of de novo biosynthesis (miRNA binding and pre-mRNA binding) (Table 2). After transcription factor enrichment analysis, the proteins that most likely regulate the observed changes in gene expression after Cr administration are involved in cell survival, apoptosis, proliferation, differentiation, migration, and the cytoskeletal structure. To highlight this, among the top ranked transcription factors (Figure 3A) of our analysis we found: (i) FOS is a G0/G1 switch regulatory protein that heterodimerizes with members of the JUN family of transcription factors to form AP-1 complexes and regulate signal transduction, cell proliferation, and differentiation [79]; (ii) SP1 was the first to be cloned and characterized of the specificity protein–Krüppel-like factor (Sp/KLF) family of transcription factors, which are involved in multitude of cellular pathways and processes by regulating tissue- and developmental-stage-specific gene expression [80]; (iii) NFYA is one of the three subunits of a highly conserved and ubiquitously expressed heterotrimeric transcription factor that regulate gene expression at promoter regions [81]; (iv) EZH2 and SUZ12 are essential for the integrity of the polycomb group complexes, the expression of which rises at the G1/S phase transition [82,83]; (v) ZMIZ1 regulates the activity of various transcription factors, including the androgen receptor, Smad3/4, and p53 [84]; and (vi) MYC is implicated in cell growth [85], proliferation, differentiation [86], and cellular adhesion and migration [87,88], and may dimerize with MAX to bind DNA and exert its effects [89].

The analysis of upstream regulatory signaling pathways resulted in MAPKs, CDKs, CSNK2A1, and Akt/PKB being the most representative after Cr administration. Although no clinical research has been conducted to study the changes in CSNK2A1 or CDK expression after Cr supplementation, one would expect a possible regulatory effect given that both proteins play an essential role during cell cycle progression and differentiation [90,91]. It is interesting to note that CK and CSNK2A1 activities vary similarly during muscle cell differentiation, with CSNK2A1 being dispensable for (i) the maintenance of the myogenic identity, (ii) the expression of early myogenic markers and late muscle-specific gene expression, and (iii) the control of myoblast fusion [92]. CSNK2 has been reported to control the Janus kinase–signal transducer and activator of transcription (JAK/STAT) signaling pathway [93], which is the principal mechanism for a wide range of growth factors and cytokines where other pathways such as MAPKs and PI3K/Akt are involved [94]. This JAK/STAT pathway activation might regulate somatic growth via binding transcriptional enhancers in the *IGF1* locus [95]. In addition, CSNK2A and CDKs share a common target, the integrator of neurotransmitters called dopamine- and cAMP-regulated phosphoprotein-32 (DARPP-32) [96]. DARPP-32 interacts with β-adducin at the cytoskeleton to mediate rapid environmental effects on neurons [97], which might explain its upregulation after Cr treatment to promote differentiation and maturation of neurons [98]. Again, we highlight the dynamic biosensor role of the CK/PCr system in this type of chemomechanical energy transduction process. Recently, CSNK2A has been reported to be a critical element of the Th17/Treg cell balance and differentiation [99], meaning more research is needed to unravel the mechanisms that explain the effects of Cr on chromatin remodeling in immune cells. Since immunity regulation goes beyond the scope of this review, please refer to [15] for a recent comprehensive summary of the current findings and future directions in this regard.

**Figure A1:**
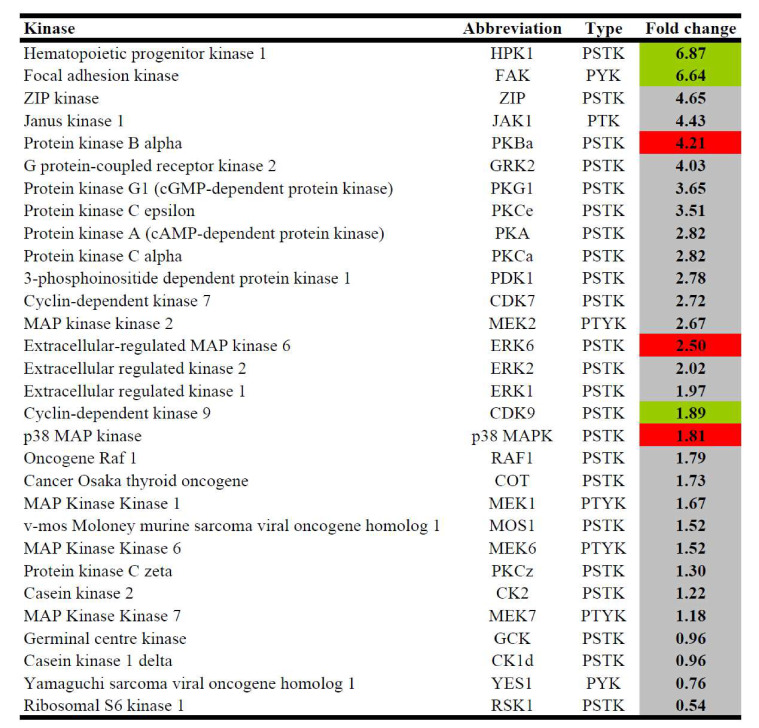
Effects of ten days of CrM supplementation in healthy young men (with no changes in their dietary intake) on protein kinase content using the Kinetworks KPKS 1.0 Protein Kinase screen. PSTK = protein–serine/threonine kinase; PTYK = protein–threonine/tyrosine kinase; PYK = protein–tyrosine kinase; PTK = protein–threonine kinase. Significant increases (red; *p* < 0.05), trends for increases (green; *p* < 0.09), and no change (grey) in protein content with CrM supplementation vs. placebo are indicated. Reproduced from Safdar et al. (2008).

The phosphorylation cascades of MAPKs are key components of extracellular signal transduction, with important roles in cell survival, proliferation, and differentiation. The signal begins at the activation of receptor tyrosine kinases (RTKs) and other transmembrane receptors via guanine nucleotide exchange factors (GEFs, such as the son of sevenless [SOS]) that lead to the active form of the small G-proteins Ras [100]. After their activation, the Ras variants interact with MAPK kinase kinases (MAPKKK, also called MAP3Ks or MEKKs, with Raf being the most representative member) to activate MAPK kinase (MAPKK, also known as MEK), then finally MAPKs through scaffold and adaptor proteins such as KSR, JIP, and OSM [101]. There are three main MAPK families with different isoforms, namely the ERKs, the JNKs, and the p38 MAPKs (p38 α/β/γ/δ) [102]. Besides the hormone regulation of RTKs (e.g., growth hormone, IGF-1), the MAPK pathway may be activated by energy-driven mechanosensing [103,104] and osmosensing [105] mechanisms. Alternatively, activation of the IGF-1 receptor may stimulate the PI3K/Akt/mTORC1 pathway, which has a crucial role in protein synthesis via RPS6K and 4E-BP1 [106]. Our functional annotation analysis indicated that G-protein-coupled receptor binding (*p* = 0.002921) and Rac GTPase binding (a subgroup of the Ras superfamily of GTP hydrolases) (*p* = 0.003855) are among the top molecular functions of the differentially expressed genes that change after Cr administration. It is possible that the experimentally reported activation of MAPKs after Cr administration might be due to mechano- and osmosensing mechanisms driven by the optimization of the CK/PCr system. For instance, CrM supplementation for ten days has resulted in the mRNA overexpression of *SPHK1* (osmosensing gene) and various MAPKs in healthy men [70] (Appendix A Figure A1).

**Figure 4:**
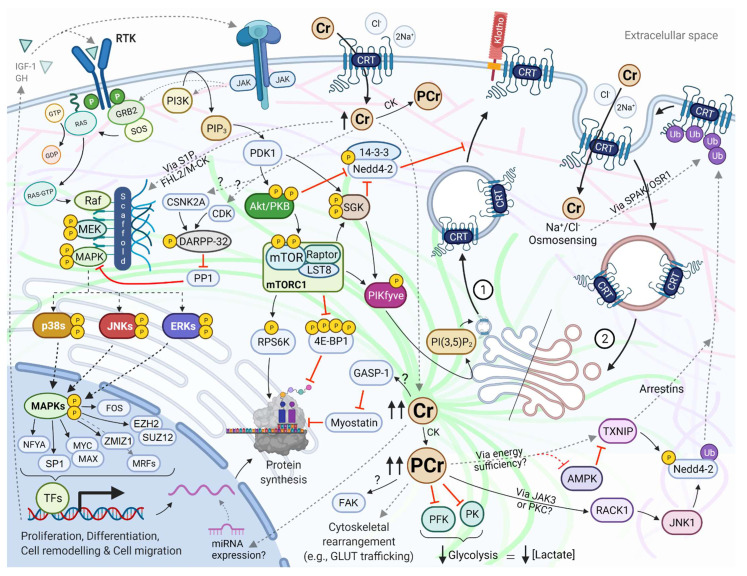
Bioinformatics- and knowledge-based pathway reconstruction after Cr supplementation. This is a representation of pathways interactions based on the results of our enrichment analysis of differentially expressed genes after increasing cellular Cr concentration and the available experimental evidence. This functional network follows the ‘bio-logic’ (integration of bottom-up and top-down directions) of the genotype–outcome interaction. MAPK activation can occur via osmosensing pathways that activate Ras/Raf (e.g., S1P/SPHK1) and mechanosensing pathways that involve mechanical and energy optimization of the cytoskeleton (e.g., Four-and-a-Half Lim 2 is an important mechanosensor that triggers hypertrophy in response to strain and also docks key metabolic enzymes involved in the energy transduction process, such as M-CK, adenylate kinase, and phosphofructokinase). Several subunits of the protein complexes and the architecture of the cytoskeleton are not depicted for readability. We cautiously suggest two dose–response functional pathways for the regulation of the Cr uptake: a kinase-driven mechanism as a result of the initial Cr-enriched environment, which is more related to the anterograde trafficking via endolysosome-specific phosphoinositide compounds (1); and a ubiquitin-driven mechanism that controls the excessive Cr uptake, which is more related to the retrograde trafficking via clathrin-dependent and clathrin-independent processes (2). Interlinking protein filaments of the cytoskeleton are represented with lighter-colored lines in the background. See the sections of the manuscript for rationale, citations, and more abbreviations. Dashed arrows represent multiple steps. AMPK: AMP-activated protein kinase; CK: creatine kinase; GASP-1: growth and differentiation factor (GDF)-associated serum protein-1; GDP: guanosine diphosphate; GH: growth hormone; GLUT: glucose transporter; GRB2: growth factor receptor-bound protein 2; GTP: guanosine triphosphate; IGF-1: insulin-like growth factor-1; LST8: target of rapamycin complex subunit LST8; MRFs: myogenic regulatory factors; Nedd4-2: E3 ubiquitin–protein ligase NEDD4-like; OSR1: oxidative-stress-responsive kinase 1; PDK1: phosphoinositide-dependent kinase-1; PFK: phosphofructokinase; PI(3,5)P_2_: phosphatidylinositol 3,5-bisphosphate; PIKfyve: 1-phosphatidylinositol 3-phosphate 5-kinase; PI3K: phosphoinositide 3-kinase; PIP_3_: phosphatidylinositol (3,4,5)-trisphosphate; PK: pyruvate kinase; SGK: serum- and glucocorticoid-regulated kinase; SPAK: SPS1-related proline–alanine-rich kinase; RACK1: receptor for activated C kinase 1; RTK: receptor tyrosine kinases; TFs: transcription factors. Source: created by the authors (D.A.B.) with BioRender—https://biorender.com/ (accessed on 10 May 2021).

Interestingly, the results of our kinase enrichment analysis and the identification of hub nodes (downstream effectors of the MAPK and IGF-1/PI3K/Akt pathways) (Figure 3) are in high agreement with the available low-throughput, high-sensitivity experimental in vitro and in vivo evidence after Cr administration, such as qRT-PCR, Western blotting, and electrophoretic mobility shift assay. Several human and animal studies have shown that Cr brings higher growth hormone concentrations [107]; overexpression of IGF-1 [34,36,108,109]; upregulation and higher activity of Akt/PKB [70,110,111]; downregulation of myostatin and increase in GASP-1 [35]; overexpression of p38α (also called MAPK14) [69,112,113]; overexpression and higher activity of RPS6K and 4E-BP1 [37,38,114]; upregulation of myocyte enhancer factor isoforms [111,115]; overexpression of myogenic regulatory factors, such as MyoD, Myogenin, Myf5, and MRF4/Myf6/Herculin [32,33,36]; and overexpression of myosin heavy chain (MHC) isoforms [116]. Remarkably, our kinase enrichment analysis (Figure 3C–D) showed high agreement with the protein kinase content after CrM supplementation, as reported by Safdar et al. (Figure A1) [70]. All aforementioned experimental evidence validates the power of the computational prediction of the multi-species convergent analysis, which highlights the need to include this before performing low-throughput experimental analysis. It must be noted that many of the signaling pathways that might be activated after Cr administration (Figure 4) follow ‘concurrent reaction with exchange motifs’, which are characterized by the high level of enzymes transferring phosphorus-containing groups (EC 2.7) [77].

### 4.2. Creatine and miRNAs

Interesting results were obtained from Enrichr using the miRTarBase data library to analyze the miRNAs interactions. Regarding cancer, recent evidence has suggested that Cr supplementation might have a carcinogenic effect [117]; nevertheless, contrary to this hypothesis, the formation of carcinogenic heterocyclic amines is unrelated to CrM supplementation [118], and even clinical research has demonstrated a potential anti-tumor progression [119]. In fact, downregulation of the CK isozymes and low levels of PCr and Cr are associated with sarcoma and adenocarcinoma progression [120]. Moreover, Cr has been reported to enhance the anti-cancer effects of methylglyoxal in chemically induced muscle cancerous cells in vitro and in sarcoma mouse cells in vivo [121]. In our bioinformatics-assisted review of the Cr metabolism [3], we discussed the observed latency towards reliance on glycolysis at high physical work rates after Cr administration, which might explain the observed reduction in lactate accumulation (possibly via inhibition of phosphofructokinase [122] and pyruvate kinase [123]) and the potential anti-tumor progression of Cr and its derivatives [124,125,126]. In this sense, the human muscle transcriptome analysis performed by Safdar et al. [70] demonstrated that a 10-day CrM supplementation period decreased the phosphofructokinase mRNA content by 21% versus placebo. For a recent review on the role of Cr in T cell anti-tumor immunity and cancer immunotherapy, please refer to Li and Yang [127].

Our enrichment analysis revealed for the first time that Cr administration might impact certain miRNAs that control cancer progression and muscle function. We are aware that more experimental evidence is needed to the identify clinical effects, therapeutic targets, and potential biomarkers in health and disease states, especially in certain cancer phenotypes where a Cr-dependent tumor progression has been proposed based on preclinical data [128]. Table 3 briefly describes the functions of several of the top-ranked miRNAs that were computationally predicted to interact with the list of selected differentially expressed genes altered after CrM administration.

Since most of the identified miRNAs repress cancer progression, it would be appropriate to investigate the effects of CrM administration on these biological elements to evaluate at which point Cr might regulate excessive proliferation. These post-transcriptional gene regulators open a new field of research regarding the therapeutic role of CrM supplementation on health-related conditions. It needs to be noted that the osmosensing activation of MAPK via Sphk1/S1P is related to several miRNAs [142], as well as the notion that the effect of Cr on focal adhesion kinase [38,70] (Figure A1) might control muscle cell differentiation through a small set of miRNAs that are connected to the focal adhesion signaling during muscle regeneration, as was reported recently [143]. Further experimental validation is warranted in the future.

### 4.3. The Regulation of the Creatine Transporter

In agreement with Santacruz et al. (2015), the elucidation for creatine transporter (CRT, also known as SLC6A8) regulation merits further study given its importance to the optimal function of several human tissues [144]. It needs to be noted that Cr belongs to a set of seven putative systems biomarkers for Alzheimer’s disease, Parkinson’s disease, and amyotrophic lateral sclerosis [145], which highlights its remarkable influence on neuron survival and function. Since it is not able to increase itself to the same extent as skeletal muscle, most of the research on enhancing Cr uptake using intermediate compounds (e.g., guanidinoacetate) or derivatives (e.g., Cr ethyl ester, dodecyl Cr ester, cyclocreatine, and Cr gluconate) is focused on the brain [18] and the heart [146]. Additionally, even if intracellular Cr levels increase after CrM (or Cr analogous) supplementation, the uptake is limited by CRT downregulation due to mechanisms that are not fully understood. CrM supplementation has been shown to reduce the maximum rate of CRT activity (V_max_) with no changes in the CRT expression [144,147,148], reinforcing the hypothesis of endosomal internalization.

Based on the results of our CFG and enrichment analysis, we performed a pathway reconstruction using well-characterized and experimentally validated PPIs to identify the possible mechanistic progression for the trafficking regulation of the CRT after CrM supplementation. By combining a knowledge-based approach [149] and the results of our upstream regulatory analysis to build the pathway, we obtained two dose–response and complementary functional networks: (i) a kinase-driven mechanism as a result of the initial Cr-enriched environment (more related to the anterograde trafficking); (ii) a ubiquitin-driven mechanism that controls the excessive Cr uptake (more related to the retrograde trafficking) (Figure 4). A third possible mechanism encompasses the initial upregulation of the full-length CRT by splice variants (SLC6A8C and SLC6A8D), considering that our functional annotation revealed that the differentially expressed genes perform a function in the U5–snRNP complex, which is involved in the pre-mRNA splicing events; however, this will not be further discussed because of the lack of experimental support beyond the study by Ndika et al. [150].

The enhancement of the CK/PCr system (Cr-enriched environment) via CrM supplementation leads to the previously discussed activation of mechano- and energy-sensing pathways, such as MAPKs and IGF-1/PI3K/Akt. The CRT function may be susceptible to regulation by several kinases of these cascades [151]. Two-electrode voltage clamp recordings have revealed that mTOR [152], SGK1/3 [153], and 1-phosphatidylinositol 3-phosphate 5-kinase (PIKfyve) [154] enhance Cr uptake. It has been suggested that this process might be mediated by the production of phosphatidylinositol-3,5-bisphosphate (PI(3,5)P_2_), which is implicated in cytoskeleton rearrangement and cellular motility and provides spatial and temporal control for membrane trafficking [155]. For example, GLUT-4 translocation is regulated by PI(3,5)P_2_ concentrations, given that PIKfyve is localized in a subpopulation of highly dynamic vesicles containing this transporter and may be activated by Akt/PKB after triggering the insulin receptor pathway [156]. Additionally, SGK can phosphorylate the E3 ubiquitin–protein ligase NEDD4-like (Nedd4-2), which reduces the ability of Nedd4-2 to interact with target proteins due to the interaction of the phosphorylated form with its scaffolding protein 14-3-3 [157]. This positive feedforward mechanism has been reported in the trafficking regulation of other membrane transporters [158,159]. Interestingly, Klotho protein, a transmembrane protein determinant of aging and life span, upregulates the activity of CRT by stabilizing the carrier protein in the cell membrane [160]. Klotho serves as a powerful regulator of cellular transport across the plasma membrane [161] and is associated with the activation of ERK 1/2 and SGK1 signaling cascades [162]. In recent years, Klotho has been linked to glycolysis inhibition and anti-cancer activity [163], which deserves more research due to its effect on Cr uptake. Further research is also needed to understand the molecular processes that account for the increased CRT expression and Cr uptake after activation of the estrogen-related receptor α [164].

As part of the hormetic dose–response, it is also plausible that the continuous increase in Cr concentration activates cellular responses that negatively regulate Cr uptake via possible ubiquitin-related mechanisms. Zervou et al. (2016) showed that very high Cr concentrations (>160 nmol·mg^−1^ protein) might lead to impaired energy metabolism in cardiomyocytes of transgenic mice overexpressing CRT, in what the authors defined as a ‘substrate-rich but energy-poor heart’ [165]; thus, similar to other protein carriers, downregulation of the CRT might result from nutrient sensitivity, energy sufficiency, and osmotic changes [166]. Thioredoxin-interacting protein (Txnip), a member of a novel family of proteins termed α-arrestin or arrestin-domain-containing proteins that possess homology to β-arrestins [167], has been identified as the only gene upregulated after saturating cells with Cr in vitro (Figure 2). This seems to be relevant in vivo, since higher mRNA (57.6%) and protein (28.7%) levels of Txnip were found in animal models overexpressing CRT, while Cr-deficient mouse hearts showed lower mRNA (39.71%) expression in comparison to wild-type animals [72]. Thioredoxin (*Txn1*) is a small and ubiquitously expressed protein, which in conjunction with thioredoxin reductase, reduces free radical oxygen species, protein disulfides, and other oxidants [168]. The Txnip binds to Txn1 to exert critical functions in terms of energy metabolism (e.g., increase redox stress, inhibit cellular glucose uptake, among others) [169]. Notably, the arrestin domains are the crucial structural elements in the metabolic functions of proteins such as Txnip [170], given that they operate as multifaceted protein trafficking adaptors that serve as signaling scaffolds of multiple protein kinases. They bind to membrane cargo proteins and interact with the adaptor protein complex 2, which is the second most abundant component of clathrin-coated vesicles, in order to promote endocytic turnover of their cargos [171]. As the archetypal β-arrestin, Tnxip has two major structural domains: the NH_2_ domain for protein–protein interaction, with SH_3_-binding proteins and MAP3Ks [172]; and the COOH domain, with proline-rich motifs that not only bind to both adaptin and clathrin heavy chains but also interact and recruit WW-domain-containing E3 ubiquitin ligases, such as Nedd4-2, to ubiquitinate proteins and promote internalization to endosomes [173]. In addition, α-arrestins are likely to utilize other mechanisms to mark cargo for internalization by clathrin-independent endocytosis [174]. It must be noted that JNK1, a top-ranked protein kinase from our enrichment analysis, may phosphorylate and activate Nedd4-2 [175,176]. Interestingly, upstream activators of JNK1 such as JAK2 [177], JAK3 [178], and PKC [179] have been reported to be negative regulators of the CRT; therefore, endosomal trafficking of CRT might be highly regulated by Cr concentration and energy sufficiency. Likewise, regulation of GLUT proteins by Txnip depends on glucose and energy-sensing pathways, taking into account that if the AMP/ATP ratio increases, AMPK becomes activated and phosphorylates Txnip to induce its degradation. This results in the repression of GLUT protein endocytosis and promotes glucose uptake to relieve energy stress [180]. Regarding Cr uptake, contradictory findings have been found regarding the regulation of CRT by AMPK [181,182], which deserves more research; the biologically conserved response to the mechanical stress induced by altered osmolarity [166] might also contribute to the control of Cr uptake (as a cellular hyperhydrating agent). Besides the kinase activity and the possible ubiquitination of CRT via Txnip/Nedd4-2, the JNK is considered the main mechanism for osmosensing signal transduction [183]. Furthermore, it has been demonstrated that SPAK and OSR1 are negative regulators of the CRT [184]. These kinases are part of the osmosensing WNK-SPAK/OSR1 pathway, considered the master regulator of cation-chloride cotransporters [185], such as the CRT. This osmosensing regulation of clathrin-mediated endocytosis is preserved among several species [186,187,188,189].

It is worth mentioning the successful strategies carried out in recent years to rescue misfolded and endoplasmic-reticulum-trapped CRT variants with the use of pharmacochaperones such as the FDA-approved 4-phenylbutyrate [190]. Several mutations of the CRT may result in transporter malfunction due to misfolding followed by impaired expression or reduced trafficking to the plasmalemma surface. Conformational changes of the CRT might trigger quality control mechanisms involving N-glycosylation (e.g., unfolded protein response) [191]. For a recent comprehensive review on this topic and novel therapeutic strategies related to Cr deficiency syndrome, please refer to [192].

## 5. Limitations, Strengths, and Future Directions

This study should be interpreted in light of the following limitations and strengths: (i) A very low number of ‘omics’ studies have evaluated the effects of CrM supplementation in humans, meaning future (epi)genomics, transcriptomics, proteomics, and metabolomics studies in this area are needed. Specifically, only one human dataset was deposited in the repositories (GEO), showing again that more publicly available datasets are needed to strengthen the current findings; however, the CFG approach has been highlighted for its ability to distinguish signals from noise, even with limited cohorts and datasets [74,193]. (ii) The findings of the CFG, the bioinformatics enrichment analysis, as well as the conclusions from in vitro and in vivo animal models should be interpreted with caution, given they might not fully reflect cellular changes in humans after CrM supplementation but rather represent a mechanistic insight into cellular dynamics and proof-of-concept evidence to develop novel therapeutic strategies through the assessment of pharmacological activators and inhibitors. (iii) Despite the limitations in the number of expression datasets, the results obtained in the enrichment analysis (Enrichr and X2K) were contrasted and supported by low-throughput, high-sensitivity experimental evidence that has identified targeted genes and proteins related to the activated pathways after CrM supplementation in humans. However, experimental validation of candidate genes, protein, and miRNA hubs from our analysis is warranted in the future. (iv) This study is a clear example of the powerful features of the ‘omics’ high-throughput technologies and bioinformatics tools and may represent a workflow for future studies that analyze emergent nutrients with potential applications in sports and health or disease. In fact, understanding the interactions between system components and their regulatory aspects allow following a ‘biologic’ interpretation that is different and much more valuable than the common top-down or bottom-up approaches.

Future studies on identifying biological regulators of CrM supplementation on health and disease include: (i) the changes in miRNAs content (and other regulator non-coding RNAs); for instance, the use of a small-interfering RNA against Txnip resulted in increased Cr uptake [72], meaning further work will contribute to elucidating the mechanisms of the Txnip–CRT interaction and its potential therapeutic use as a next-generation medicine [194]; (ii) the pharmacochaperones (e.g., 4-phenylbutyrate) and their safety and efficacy to treat pathologies associated with the Cr deficiency syndrome; (iii) the implementation of a systems biology approach as a necessary and unavoidable process to study other metabolic networks of high complexity, such as the Cr metabolism; (iv) the integration of (epi)genomics, transcriptomics, phosphoproteomics, and metabolomics analyses (multi-omic analysis). In this sense, a very recent tool called Causal Oriented Search of Multi-Omic Space (COSMOS) [195] was developed to extract mechanistic insights in a more consistent and robust manner. This opens up an exciting field of research with multiple applications in several human conditions.

## 6. Conclusions

The CK/PCr system acts as a hub for chemomechanical energy transduction (i.e., dynamic biosensor) during the cellular allodynamic states. For the first time, a CFG with enrichment analysis was performed to identify relevant pathways and biological processes mediating the effects of Cr in health and disease. The results of our secondary analysis of available gene expression data showed that several cytoskeleton-mediated, energy-driven, mechanoadaptative processes possibly account for the wide range of effects and diseases after alterations of intracellular Cr concentrations. Additionally, we cautiously suggest two dose–response and complementary functional networks for the negative regulation of CRT after the continuous increase in Cr concentration: (i) a kinase-driven mechanism responsible for anterograde trafficking during the initial Cr-enriched environment; (ii) a ubiquitin-driven mechanism that controls the excessive Cr uptake. In general, Cr metabolism encompasses one of the most complex and dynamic networks, meaning further studies will be expected to outline evidence detailing the positive roles of CrM supplementation in other uncovered areas of health and disease. This work might contribute to a better understanding of the well-reported benefits of Cr in sports and its potential in health and disease, although more research is warranted to validate some of the proposed mechanisms.

## Figures and Tables

**Table 1:** Datasets used in the convergent functional genomic analysis.

Organism	Reference	GEO Number	Design	Creatine	Control	Platform
Human	[70]	GSE7877	Expression profiling of *vastus lateralis* muscle in a randomized, placebo- controlled, crossover, double-blind design in young, healthy, non-obese men supplemented with CrM vs. placebo (dextrose) for ten days	12	12	Buck Institute_Homo sapiens_25K_verC
Mouse	[71]	GSE5140	Analysis of brains of C57Bl/6J animals fed a Cr-supplemented diet for six months	6	7	Affymetrix Mouse Genome 430 2.0 Array
Mouse	[72]	GSE42356	3T3 fibroblasts overexpressing CRT were treated with 5mM CrM	3	3	Illumina MouseWG-6 v2.0 expression beadchip

**Table 2:** Functional annotation and miRNA enrichment analysis of the list of genes from the convergent analysis.

Category	GO ID	Term	Adjusted *p*-Value
Biological Process	GO:0030879	Mammary gland development	0.00538
	GO:0043069	Negative regulation of programmed cell death	0.01899
	GO:0045765	Regulation of angiogenesis	0.03483
	GO:0071542	Dopaminergic neuron differentiation	0.05505
	GO:0001934	Positive regulation of protein phosphorylation	0.05505
Cellular Component	GO:0005664	Nuclear origin of replication recognition complex	0.2386
	GO:0005682	U5 snRNP	0.2386
	GO:0031904	Endosome lumen	0.2386
	GO:0046540	U4/U6 x U5 tri-snRNP complex	0.2386
	GO:0005637	Nuclear inner membrane	0.2386
Molecular Function	GO:0035198	miRNA binding	0.05290
	GO:0036002	Pre-mRNA binding	0.05290
	GO:0048365	Rac GTPase binding	0.05290
	GO:0031593	Polyubiquitin-modification-dependent protein binding	0.05290
	GO:0001664	G-protein-coupled receptor binding	0.05290
**Database**	**miRBase Accession**	**Description**	**Adjusted *p*-Value**
miRTarBase	MIMAT0000416	Mature sequence *Homo sapiens* miR-1-3p	0.0942
	MIMAT0000275	Mature sequence *Homo sapiens* miR-218-5p	0.1405
	MIMAT0000447	Mature sequence *Homo sapiens* miR-134-5p	0.1513
	MIMAT0022487	Mature sequence *Homo sapiens* miR-5694	0.1961
	MI0000542	Stem loop sequence *Homo sapiens* miR-320a	0.2492

**Table 3:** Information of predicted miRNAs interacting with the list of genes from the CFG analysis.

MicroRNA	Relevant Information
miR-1-3p	Suppresses the proliferation of hepatocellular carcinoma [129] and slows the proliferation and invasion of gastric [130] and lung adenocarcinoma [131].
miR-218-5p	Significantly upregulated during myogenic differentiation after activating the IGF-1 and MAPK/ERK pathways [132].
miR-134-5p	Lower levels are found in prostate cancer compared to benign prostatic hyperplasia [133]. In addition, it might have neuroprotective effects by regulating the miR-134-5p/CREB pathway in both humans and mice [134].
miR-5694	Mediates downregulation of AF9 (a subunit of the super elongation complex and associates with the histone methyltransferases) and provides metastatic advantages in basal-like breast cancer cells [135].
miR-320a	Although associated with certain types of cancer, it has been shown to inhibit the proliferation and progression of melanoma [136] and gastric adenocarcinoma [137].
miR-200b-3p	Higher expression is found in prostate cancer compared to benign prostatic hyperplasia [133].
miR-126a-3p	It targets low-density lipoprotein-receptor-related protein 1 and blocks WNT signaling, which partially explain the anti-tumor effects of curcumin [138].
miR-378a-3p	Exhibits tumor-suppressive and anti-metastatic effects in esophageal squamous cell carcinoma [139] and glioblastoma multiforme [140]; however, miR-378a might also have a pro-angiogenic effect on myoblasts and control vascularization of skeletal muscle [141].

## Data Availability

The datasets supporting the reported results in this article are available in the GEO (Gene Expression Omnibus) repository (https://www.ncbi.nlm.nih.gov/geo/): GSE7877 (https://www.ncbi.nlm.nih.gov/geo/query/acc.cgi?acc=GSE7877), GSE5140 (https://www.ncbi.nlm.nih.gov/geo/query/acc.cgi?acc=GSE5140), GSE42356 (https://www.ncbi.nlm.nih.gov/geo/query/acc.cgi?acc=GSE42356). All datasets were accessed on 14 January 2021.

## Supplementary Materials

The following are available online at https://www.mdpi.com/article/10.3390/nu13082521/s1, Table S1: GSE5140 Top genes, Table S2: GSE42356 Top genes, Table S3: GSE7877 Top genes.
